# Crystal structure and Hirshfeld surface analysis of the anionic tetra­kis-complex of lanthanum(III) NMe_4_La*L*_4_ with the CAPh-ligand dimeth­yl (2,2,2-tri­chloro­acet­yl)phospho­ramidate

**DOI:** 10.1107/S2056989021011750

**Published:** 2021-11-16

**Authors:** Mariia B. Struhatska, Nataliia S. Kariaka, Vladimir M. Amirkhanov, Viktoriya V. Dyakonenko, Maksym Seredyuk

**Affiliations:** a Department of Chemistry, Taras Shevchenko National University of Kyiv, Volodymyrska Street 64, Kyiv 01601, Ukraine; b SSI "Institute for Single Crystals" of National Academy of Sciences of Ukraine, Nauky ave. 60, 61001 Kharkiv, Ukraine

**Keywords:** crystal structure, lanthanum(III) complex, β-diketone derivatives, carbacyl­amido­phopsphates, rare earth metals, coordination compound, Hirshfeld analysis

## Abstract

The anionic tetra­kis-complex of lanthanum(III) NMe_4_La*L*_4_ with the CAPh-ligand dimethyl (2,2,2-tri­chloro­acet­yl)phospho­ramidate (H*L*), namely, tetra­methyl­ammonium tetra­kis­{2,2,2-tri­chloro-1-[(di­meth­oxy­phosphor­yl)imino]­ethano­lato}lanthanum(III), (C_4_H_12_N)[La(C_4_H_6_Cl_3_NO_4_P)_4_], has been synthesized, crystallized and structurally characterized by X-ray diffraction. The lanthanide ion is surrounded by four anionic, bis-chelating CAPh ligands forming the complex anion with a coordination number of eight for La^3+^ and NMe_4_^+^ as the counter-ion. The coordination polyhedron of the La^3+^ ion was inter­preted as a triangular dodeca­hedron.

## Abstract 2

A crystal structure of the anionic tetra­kis-complex of lanthanum(III) NMe_4_La*L*_4_ with the CAPh-ligand dimethyl (2,2,2-tri­chloro­acet­yl)phospho­ramidate is reported and discussed.

## Chemical context

Considerable inter­est in the luminescence properties of lanthanide coordination compounds results from their potential applications in modern technologies and medicine (Eliseeva *et al.*, 2010 ▸; Kido *et al.*, 2002 ▸; Tsukube *et al.*, 2002 ▸). In particular, use of P,N-substituted analogues of β-diketone such as carbacyl­amido­phopsphates (CAPh) (Amirkhanov *et al.*, 2014 ▸) with the C(O)NHP(O) structural fragment as ligands is promising because of their powerful chelating properties (Skopenko *et al.*, 2004 ▸; Amirkhanov *et al.*, 2014 ▸) and ability to sensitize the luminescence of lanthanides (Kariaka *et al.*, 2016 ▸; Pham *et al.*, 2017 ▸; Kariaka *et al.*, 2018 ▸). In this work, the synthesis and crystal structure of the anionic tetra­kis-complex of lanthanum(III) containing the CAPh-ligand dimethyl (2,2,2-tri­chloro­acet­yl)phospho­ramidate and a tetra­methyl­ammonium cation (formula NMe_4_La*L*_4_) is reported. 
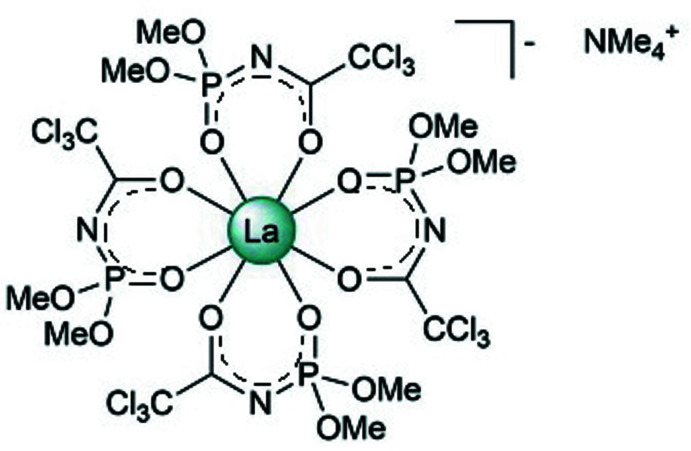


## Structural commentary

**Figure 1:**
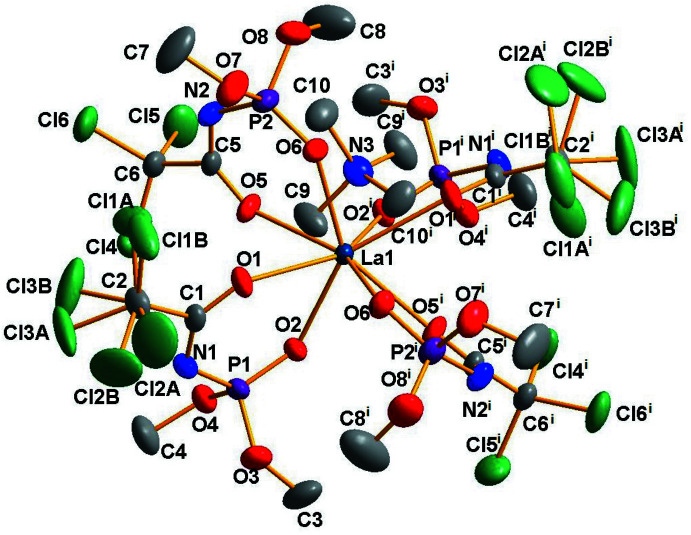
The mol­ecular structure of the title compound with displacement ellipsoids drawn at the 25% probability level. Hydrogen atoms are omitted for clarity [Symmetry code: (i) −*x* + 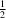 , *y*, −*z* + 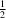 ).

The title compound (C_4_H_12_N)[La(C_4_H_6_Cl_3_NO_4_P)_4_] crystallizes in the monoclinic crystal system with two mol­ecules in the unit cell. Both the cation and the anion have crystallographically-imposed *C*_2_ symmetry with atoms La1 and N3 located on the twofold axis. The mol­ecular structure of the complex is shown in Fig. 1 ▸. In the complex, the La^3+^ ion has a triangular dodeca­hedral coordination environment formed by the eight O atoms of the bidentate CAPh ligands and the N(CH_3_)_4_^+^ unit acts as the counter-ion (Fig. 1 ▸). The average La—O bond length is 2.494 Å while the La—O(C) bond lengths [2.534 (3)–2.566 (3) Å] are all longer than the La—O(P) bonds [2.432 (3)–2.445 (3) Å]. Deprotonation of the ligands leads to increasing π-conjugation in the chelating fragment and results in the bond-length changes. The C—O and P—O bond lengths are in the ranges 1.225 (5)–1.240 (6) Å and 1.475 (3)–1.476 (4) Å, respectively, with corresponding average values of 1.233 and 1.476 Å. The corresponding bond lengths in the neutral ligand H*L* are 1.202 (2) and 1.459 (2) Å (Amirkhanov *et al.*, 2014 ▸). The C—O and P—O bonds of the ligand in the complex are longer than those in the neutral ligand (H*L*), indicating greater C=O and P=O double-bond character in H*L* than in NMe_4_La*L*_4_. The C—N and P—N bonds, with lengths in the ranges 1.291 (6)–1.292 (6) and 1.598 (4)–1.602 (5) Å, respectively, in NMe_4_La*L*_4_ are shorter compared to those in the free ligand, in which the reported C—N bond length is 1.347 (2) Å and P—N is 1.676 (1) Å (Amirkhanov *et al.*, 1995 ▸).

## Supra­molecular features

**Figure 2:**
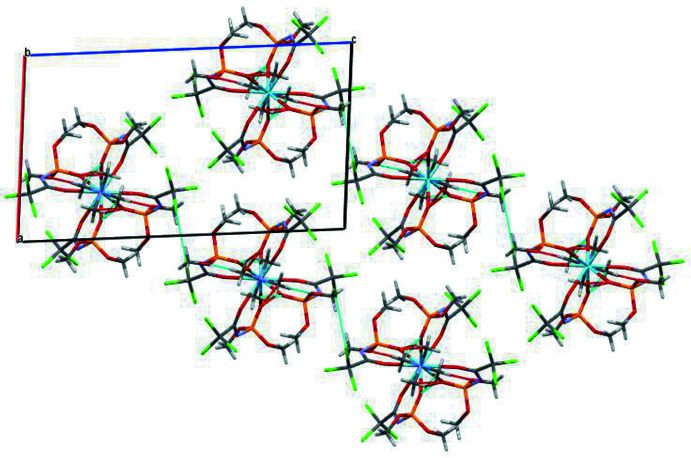
The crystal packing of the title compound viewed along the *b*-axis direction.

There are no classical hydrogen bonds in the crystal structure of the title compound, although the complexes are linked *via* numerous weak C—H⋯O and Cl⋯Cl inter­molecular inter­actions (Table 1 ▸). In particular, the PO and OCH_3_ groups of the ligands are involved in the formation of inter­actions with the hydrogen atoms of the tetra­methyl­ammonium cation, linking the complex anion and the counter-ion in a chain along the *b*-axis direction. The Cl12*A*⋯Cl12*A* ^ii^ [symmetry code (ii): −*x*, −*y* + 1, −*z* + 1] inter­actions, at 3.475 (12) Å, are only 0.03 Å less than the sum of the van der Waals radii but definitely below the maximum separation (4.0 Å) considered to represent at least weak, attractive Cl⋯Cl inter­actions (Capdevila-Cortada *et al.*, 2016 ▸). These serve to connect neighbouring chains. The crystal packing of the title compound is shown in Fig. 2 ▸.

## Hirshfeld surface analysis and fingerprint plots

**Figure 3:**
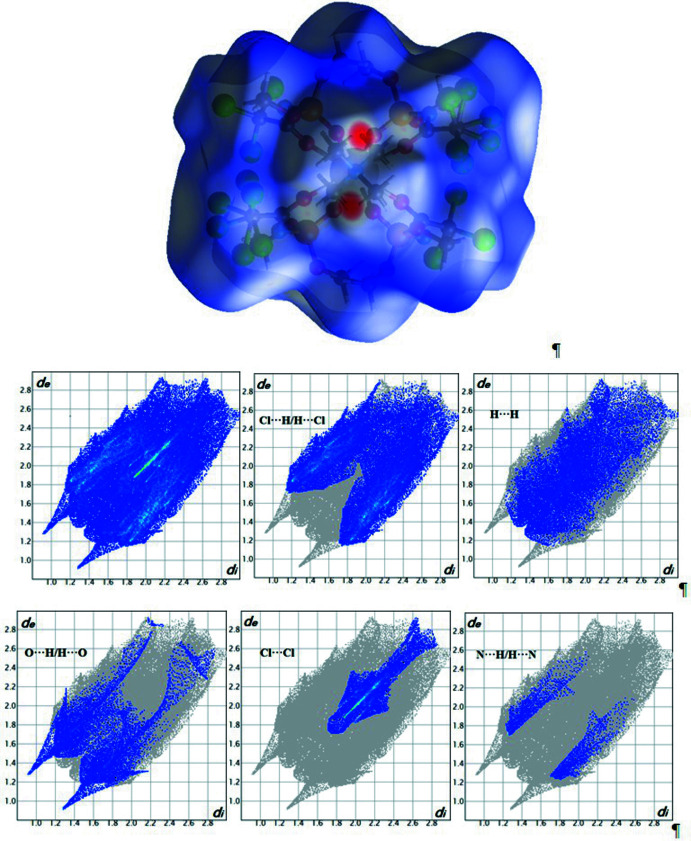
The Hirshfeld surface mapped over *d*_norm_ and two-dimensional fingerprint plots for the Cl⋯H/H⋯Cl (50.7%), H⋯H (20.8%), O⋯H/H⋯O (13.6%), Cl⋯Cl (11.6%) and N⋯H/H⋯N (3.1%) inter­actions in NMe_4_[La*L*_4_].

To visualize the inter­molecular inter­actions in the title compound, the Hirshfeld surface and its corresponding two-dimensional fingerprint plots (Spackman *et al.*, 2009 ▸) were calculated using *CrystalExplorer*17 (Turner *et al.*, 2017 ▸). There are several light-red spots on the *d_norm_* surface (Fig. 3 ▸), which correspond to O⋯H/H⋯O contacts. They are located near the oxygen atoms of the ligand PO groups and the hydrogen atoms of the tetra­methyl­ammonium cation. Thus, the strongest contacts in the crystal of the title compound exist between the NMe_4_^+^ cation and the complex anion.

The two-dimensional fingerprint plots show distances from the Hirshfeld surface to the nearest exterior atom (*d*_e_ plots) and from an inter­ior atom to the surface (*d*_i_ plots), specify atom⋯atom contacts in a crystal and provide a qu­anti­tative idea of the types of inter­molecular contacts experienced by mol­ecules. An analysis of the fingerprint plots (Fig. 3 ▸) shows that the Cl⋯H/H⋯Cl contacts make the major contribution to the Hirshfeld surface at 50.7%. The closest Cl⋯H/H⋯Cl contact occurs at *d*_i_ = *d*_e_ = 2.9 Å. The next largest contributions come from H⋯H contacts (20.8%), O⋯H/H⋯O contacts (13.6%) and Cl⋯Cl contacts (11.6%). The closest O⋯H/H⋯O contact occurs at *d*_i_ = *d*_e_= 1.35 Å. The smallest percentage contributions to the Hirshfeld surface come from the N⋯H/H⋯N (3,1%), Cl⋯O/O⋯Cl (0.1%) and O⋯O (0.1%) inter­atomic contacts.

## Database survey

A search of the Cambridge Structural Database (CSD, Version 5.41, update of March 2020; Groom *et al.*, 2016 ▸) for lanthanide complexes containing bidentate-coordinated CAPh ligands yielded 48 hits. Eight of them are tetra­kis complexes Cat[*Ln*(CAPh)_4_] of which five crystallize with two tetra­kis complexes in the asymmetric unit. Using *SHAPE* analysis (*SHAPE2.1*; Llunell *et al.*, 2013 ▸), the nine coordination polyhedra have been inter­preted as square anti­prismatic (*D*4*d*) and, for the other polyhedra, as triangular dodeca­hedral (*D*2*d*).

No CAPh-based lanthanum tetra­kis complexes have been reported to date. However, seven lanthanum complexes containing CAPhs coordinated in a bidentate manner are known. The average La—O(C) bond length is 2.411 Å while the average La—O(P) bond length is 2.351 Å. Only one tetra­kis complex based on dimethyl (2,2,2-tri­chloro­acet­yl)phospho­ramidate (NaEr*L*_4_) has been reported to date. The lengths of the CO, PO, PN and CN bonds in this complex are in the ranges 1.206–1.335, 1.422–1.489, 1.565–1.608 and 1.250–1.334 Å, respectively.

## Synthesis and crystallization

The ^1^H NMR spectrum of a solution of the title compound in DMSO-*d*_6_ was recorded on a Varian 400 NMR spectrometer at room temperature. The infrared (IR) spectrum was recorded on a Perkin–Elmer BX-II Bruker spectrometer using a KBr pellet.

**Preparation of NMe_4_La** ***L*** **_4._** LaCl_3_·7H_2_O (0.0371 g, 0.1 mmol) in the presence of HC(OC_2_H_5_)_3_ (0.14 ml, 0.7 mmol) as dehydrating agent was dissolved in 2-propanol under heating. In a separate flask, Na*L* (0.1122 g, 0.4 mmol) was dissolved in acetone and NMe_4_Cl (0.0121 g, 0.11 mmol) was added under stirring and heating. The two mixtures were combined and boiled for a minute, then cooled to room temperature. A white precipitate of NaCl was formed and was filtered off and the filtrate left in a flask in a desiccator over CaCl_2_. After two days, colourless crystals suitable for X-ray diffraction studies were obtained. The crystals were filtered off, washed with 2-propanol and dried in air.

IR (KBr pellet, cm^−1^): 2954 [*w*, ν(C—H_aliph_)], 1614 [*s*, ν(C=O)],1487 (*w*), 1367 [*s*, ν(C—N)], 1187 [*m*, ρ(CH_3_)], 1158 [*s*, ν(P=O)], 1042 [*s*, δ(POC)], 1011 (*m*), 880 (*s*), 846 (*m*), 822 (*m*), 781 (*w*), 722 (*m*), 677 [*m*, ν(CCl)], 548 [*m*, δ(PNC)], 502 (*m*).

^1^H NMR (400 MHz, DMSO-*d*_6_, 293 K): 3.61, 3.59 (*d*, 24H, CH_3_ [*L*]^−^), 3.18 (*s*, 12H, CH_3_ [NMe_4_]^+^).

## Refinement

Crystal data, data collection and structure refinement details are summarized in Table 2 ▸. The C-bound H atoms were placed in calculated positions and refined with a riding model: C—H = 0.96 Å with *U*_iso_(H) = 1.5*U*_eq_(C).

The structure exhibits disorder of the Cl atoms of one CCl_3_ substituent. All Cl—C bond distances were restrained to be similar to each other (within a standard deviation of 0.005 Å) with a target value of 1.745 Å. *U* ^ij^ values of the disordered chlorine atoms were restrained to be similar to each other (within a standard deviation of 0.02 Å^2^). The disorder ratio is 50 to 50.

## Supplementary Material

Crystal structure: contains datablock(s) I. DOI: 10.1107/S2056989021011750/mw2180sup1.cif

Structure factors: contains datablock(s) I. DOI: 10.1107/S2056989021011750/mw2180Isup2.hkl

Supporting information file. DOI: 10.1107/S2056989021011750/mw2180Isup3.cdx

Supporting information file. DOI: 10.1107/S2056989021011750/mw2180Isup4.cdx

CCDC reference: 2120335

Additional supporting information:  crystallographic information; 3D view; checkCIF report

## Figures and Tables

**Table 1:** Hydrogen-bond geometry (Å, °)

*D*—H⋯*A*	*D*—H	H⋯*A*	*D*⋯*A*	*D*—H⋯*A*
C9—H9*A*⋯O6	0.96	2.35	3.184 (7)	145
C10—H10*C*⋯O2^i^	0.96	2.33	3.218 (8)	154

Symmetry code: (i) -x+{\script{1\over 2}}, y+1, -z+{\script{3\over 2}}.

**Table 2:** Experimental details

Crystal data	
Chemical formula	(C_4_H_12_N)[La(C_4_H_6_Cl_3_NO_4_P)_4_]
*M*_r_	1290.73
Crystal system, space group	Monoclinic, *P*2/*n*
Temperature (K)	294
*a*, *b*, *c* (Å)	12.1452 (4), 10.2003 (4), 21.2846 (7)
β (°)	94.521 (3)
*V* (Å^3^)	2628.64 (15)
*Z*	2
Radiation type	Mo *K*α
μ (mm^−1^)	1.60
Crystal size (mm)	0.6 × 0.4 × 0.2

Data collection	
Diffractometer	Agilent Technologies Xcalibur, Sapphire3
Absorption correction	Multi-scan (*CrysAlis PRO*; Agilent, 2014 ▸)
*T*_min_, *T*_max_	0.694, 1.000
No. of measured, independent and observed [*I* > 2σ(*I*)] reflections	22447, 6050, 4597
*R*_int_	0.070
(sin θ/λ)_max_ (Å^−1^)	0.650

Refinement	
*R*[*F* ^2^ > 2σ(*F* ^2^)], *wR*(*F* ^2^), *S*	0.058, 0.134, 1.01
No. of reflections	6050
No. of parameters	296
No. of restraints	73
H-atom treatment	H-atom parameters constrained
Δρ_max_, Δρ_min_ (e Å^−3^)	0.92, −0.91

Computer programs: *CrysAlis PRO* (Agilent, 2014 ▸), *SHELXT* (Sheldrick, 2015*a* ▸), *SHELXL2018/3* (Sheldrick, 2015*b* ▸) and *OLEX2* (Dolomanov *et al.*, 2009 ▸).

## supplementary crystallographic information

### Crystal data

**Table:**

(C_4_H_12_N)[La(C_4_H_6_Cl_3_NO_4_P)_4_]	*F*(000) = 1280
*M**_r_* = 1290.73	*D*_x_ = 1.631 Mg m^−^^3^
Monoclinic, *P*2/*n*	Mo *K*α radiation, λ = 0.71073 Å
*a* = 12.1452 (4) Å	Cell parameters from 3067 reflections
*b* = 10.2003 (4) Å	θ = 3.2–23.6°
*c* = 21.2846 (7) Å	µ = 1.60 mm^−^^1^
β = 94.521 (3)°	*T* = 294 K
*V* = 2628.64 (15) Å^3^	Block, colourless
*Z* = 2	0.6 × 0.4 × 0.2 mm

### Data collection

**Table:**

Agilent Technologies Xcalibur, Sapphire3 diffractometer	6050 independent reflections
Radiation source: Enhance (Mo) X-ray Source	4597 reflections with *I* > 2σ(*I*)
Graphite monochromator	*R*_int_ = 0.070
Detector resolution: 16.1827 pixels mm^-1^	θ_max_ = 27.5°, θ_min_ = 3.2°
ω scans	*h* = −15→15
Absorption correction: multi-scan (CrysAlisPro; Agilent, 2014)	*k* = −13→12
*T*_min_ = 0.694, *T*_max_ = 1.000	*l* = −25→27
22447 measured reflections	

### Refinement

**Table:**

Refinement on *F*^2^	Primary atom site location: structure-invariant direct methods
Least-squares matrix: full	Hydrogen site location: inferred from neighbouring sites
*R*[*F*^2^ > 2σ(*F*^2^)] = 0.058	H-atom parameters constrained
*wR*(*F*^2^) = 0.134	*w* = 1/[σ^2^(*F*_o_^2^) + (0.057*P*)^2^ + 0.0726*P*] where *P* = (*F*_o_^2^ + 2*F*_c_^2^)/3
*S* = 1.01	(Δ/σ)_max_ < 0.001
6050 reflections	Δρ_max_ = 0.92 e Å^−^^3^
296 parameters	Δρ_min_ = −0.91 e Å^−^^3^
73 restraints	

### Special details

**Table:**

Geometry. All esds (except the esd in the dihedral angle between two l.s. planes) are estimated using the full covariance matrix. The cell esds are taken into account individually in the estimation of esds in distances, angles and torsion angles; correlations between esds in cell parameters are only used when they are defined by crystal symmetry. An approximate (isotropic) treatment of cell esds is used for estimating esds involving l.s. planes.

### Fractional atomic coordinates and isotropic or equivalent isotropic displacement parameters (Å^2^)

**Table:**

	*x*	*y*	*z*	*U*_iso_*/*U*_eq_	Occ. (<1)
La1	0.250000	0.21428 (4)	0.750000	0.03981 (13)	
Cl1A	0.3621 (6)	0.4311 (8)	0.5516 (5)	0.161 (4)	0.5
Cl1B	0.3145 (7)	0.4844 (6)	0.5495 (4)	0.144 (3)	0.5
Cl2A	0.1378 (7)	0.4598 (8)	0.5184 (4)	0.192 (4)	0.5
Cl2B	0.1311 (6)	0.3878 (10)	0.4787 (4)	0.198 (4)	0.5
Cl3A	0.2545 (8)	0.2694 (7)	0.4592 (2)	0.173 (4)	0.5
Cl3B	0.3288 (8)	0.2511 (8)	0.4835 (4)	0.189 (4)	0.5
Cl4	0.45286 (16)	−0.0370 (2)	0.58761 (10)	0.1269 (9)	
Cl5	0.63096 (17)	−0.0478 (2)	0.68243 (11)	0.1275 (8)	
Cl6	0.63761 (15)	0.1310 (2)	0.57995 (9)	0.1166 (7)	
P1	0.12224 (10)	0.04178 (13)	0.61305 (6)	0.0523 (3)	
P2	0.51080 (11)	0.38211 (15)	0.72817 (7)	0.0647 (4)	
O1	0.2329 (3)	0.3040 (3)	0.63703 (15)	0.0652 (10)	
O2	0.1564 (3)	0.0524 (3)	0.68093 (14)	0.0552 (8)	
O3	−0.0061 (3)	0.0420 (4)	0.60037 (19)	0.0790 (11)	
O4	0.1617 (3)	−0.0966 (3)	0.59196 (15)	0.0657 (9)	
O5	0.4069 (3)	0.1198 (3)	0.69257 (18)	0.0656 (10)	
O6	0.4008 (3)	0.3734 (3)	0.75292 (16)	0.0596 (9)	
O7	0.5136 (4)	0.5137 (4)	0.6907 (2)	0.0980 (14)	
O8	0.6052 (4)	0.4070 (6)	0.7800 (2)	0.1051 (15)	
N1	0.1613 (4)	0.1495 (4)	0.56501 (18)	0.0629 (11)	
N2	0.5497 (3)	0.2658 (5)	0.6845 (2)	0.0689 (13)	
C1	0.2094 (4)	0.2568 (5)	0.5840 (2)	0.0564 (13)	
C2	0.2429 (3)	0.3432 (4)	0.52989 (18)	0.0770 (17)	
C3	−0.0712 (5)	−0.0440 (9)	0.6346 (4)	0.134 (3)	
H3A	−0.056390	−0.133010	0.623339	0.201*	
H3B	−0.148061	−0.025051	0.624583	0.201*	
H3C	−0.053309	−0.031932	0.678933	0.201*	
C4	0.1430 (7)	−0.1401 (7)	0.5277 (3)	0.106 (2)	
H4A	0.176063	−0.079196	0.500412	0.159*	
H4B	0.064984	−0.144940	0.516385	0.159*	
H4C	0.175366	−0.225158	0.523438	0.159*	
C5	0.4942 (4)	0.1588 (5)	0.6742 (2)	0.0566 (12)	
C6	0.5510 (4)	0.0575 (6)	0.6321 (3)	0.0686 (15)	
C7	0.6023 (7)	0.5480 (8)	0.6526 (5)	0.153 (4)	
H7A	0.611203	0.480202	0.622176	0.230*	
H7B	0.669571	0.557443	0.679135	0.230*	
H7C	0.585323	0.629264	0.631261	0.230*	
C8	0.6282 (9)	0.3183 (11)	0.8254 (6)	0.193 (5)	
H8A	0.607447	0.232728	0.809925	0.289*	
H8B	0.587835	0.338915	0.861107	0.289*	
H8C	0.705986	0.319673	0.837739	0.289*	
N3	0.250000	0.7094 (6)	0.750000	0.0726 (18)	
C9	0.2854 (6)	0.6266 (6)	0.8055 (3)	0.103 (2)	
H9A	0.346838	0.573073	0.795873	0.154*	
H9B	0.225194	0.571408	0.815623	0.154*	
H9C	0.306877	0.681805	0.840903	0.154*	
C10	0.3433 (5)	0.7929 (6)	0.7331 (4)	0.098 (2)	
H10A	0.322433	0.839259	0.694795	0.148*	
H10B	0.406334	0.738757	0.727330	0.148*	
H10C	0.361400	0.854619	0.766399	0.148*	

### Atomic displacement parameters (Å^2^)

**Table:**

	*U*^11^	*U*^22^	*U*^33^	*U*^12^	*U*^13^	*U*^23^
La1	0.0393 (2)	0.0445 (2)	0.0364 (2)	0.000	0.00772 (15)	0.000
Cl1A	0.155 (5)	0.215 (8)	0.110 (4)	−0.118 (6)	−0.006 (4)	0.053 (6)
Cl1B	0.275 (9)	0.089 (3)	0.070 (3)	−0.080 (5)	0.027 (5)	0.002 (3)
Cl2A	0.251 (8)	0.159 (6)	0.163 (6)	0.071 (6)	0.005 (5)	0.103 (5)
Cl2B	0.185 (6)	0.237 (9)	0.158 (6)	−0.055 (6)	−0.075 (5)	0.138 (6)
Cl3A	0.343 (11)	0.137 (5)	0.049 (2)	−0.122 (6)	0.069 (4)	−0.026 (3)
Cl3B	0.271 (9)	0.138 (5)	0.183 (7)	0.040 (6)	0.173 (7)	0.041 (5)
Cl4	0.0931 (12)	0.178 (2)	0.1139 (15)	−0.0351 (13)	0.0339 (12)	−0.0808 (15)
Cl5	0.1168 (15)	0.1399 (18)	0.1283 (17)	0.0669 (14)	0.0249 (13)	0.0000 (14)
Cl6	0.1028 (13)	0.1584 (19)	0.0973 (13)	−0.0220 (12)	0.0623 (11)	−0.0242 (13)
P1	0.0515 (7)	0.0621 (8)	0.0423 (7)	−0.0098 (6)	−0.0031 (6)	0.0026 (6)
P2	0.0508 (7)	0.0657 (9)	0.0790 (10)	−0.0134 (6)	0.0141 (7)	−0.0072 (8)
O1	0.099 (3)	0.061 (2)	0.0360 (18)	−0.0053 (19)	0.0088 (19)	−0.0010 (16)
O2	0.067 (2)	0.058 (2)	0.0399 (17)	−0.0198 (16)	−0.0003 (16)	0.0034 (15)
O3	0.052 (2)	0.099 (3)	0.084 (3)	−0.005 (2)	−0.007 (2)	0.010 (2)
O4	0.081 (2)	0.067 (2)	0.0484 (19)	0.0014 (19)	0.0001 (18)	−0.0004 (17)
O5	0.059 (2)	0.062 (2)	0.082 (3)	−0.0047 (17)	0.0393 (19)	−0.0126 (19)
O6	0.0552 (19)	0.056 (2)	0.069 (2)	−0.0110 (16)	0.0149 (17)	−0.0134 (17)
O7	0.105 (3)	0.070 (3)	0.126 (4)	−0.009 (2)	0.050 (3)	0.010 (3)
O8	0.072 (3)	0.131 (4)	0.111 (4)	−0.033 (3)	−0.003 (3)	−0.023 (3)
N1	0.077 (3)	0.069 (3)	0.042 (2)	−0.019 (2)	−0.005 (2)	0.007 (2)
N2	0.049 (2)	0.078 (3)	0.082 (3)	−0.010 (2)	0.022 (2)	−0.014 (3)
C1	0.064 (3)	0.062 (3)	0.043 (3)	0.007 (3)	0.010 (2)	0.013 (2)
C2	0.109 (5)	0.073 (4)	0.048 (3)	−0.009 (4)	0.004 (3)	0.012 (3)
C3	0.063 (4)	0.194 (9)	0.143 (7)	−0.033 (5)	0.006 (5)	0.043 (7)
C4	0.154 (7)	0.105 (6)	0.057 (4)	0.012 (5)	0.005 (4)	−0.018 (4)
C5	0.049 (3)	0.076 (4)	0.047 (3)	0.003 (3)	0.016 (2)	−0.004 (3)
C6	0.053 (3)	0.094 (4)	0.062 (3)	0.000 (3)	0.019 (3)	−0.012 (3)
C7	0.148 (8)	0.114 (7)	0.212 (11)	−0.012 (6)	0.101 (8)	0.048 (7)
C8	0.168 (9)	0.215 (11)	0.183 (10)	−0.071 (8)	−0.057 (8)	0.081 (9)
N3	0.095 (5)	0.044 (3)	0.078 (4)	0.000	0.004 (4)	0.000
C9	0.162 (7)	0.071 (4)	0.071 (4)	0.034 (5)	−0.016 (4)	−0.008 (3)
C10	0.100 (5)	0.073 (4)	0.127 (6)	−0.009 (4)	0.035 (5)	−0.027 (4)

### Geometric parameters (Å, º)

**Table:**

La1—O1	2.566 (3)	O7—C7	1.442 (7)
La1—O1^i^	2.566 (3)	O8—C8	1.337 (9)
La1—O2	2.432 (3)	N1—C1	1.291 (6)
La1—O2^i^	2.432 (3)	N2—C5	1.292 (6)
La1—O5^i^	2.534 (3)	C1—C2	1.531 (6)
La1—O5	2.534 (3)	C3—H3A	0.9600
La1—O6	2.445 (3)	C3—H3B	0.9600
La1—O6^i^	2.445 (3)	C3—H3C	0.9600
Cl1A—C2	1.735 (5)	C4—H4A	0.9600
Cl1B—C2	1.716 (5)	C4—H4B	0.9600
Cl2A—C2	1.747 (4)	C4—H4C	0.9600
Cl2B—C2	1.733 (4)	C5—C6	1.564 (7)
Cl3A—C2	1.698 (4)	C7—H7A	0.9600
Cl3B—C2	1.764 (4)	C7—H7B	0.9600
Cl4—C6	1.751 (6)	C7—H7C	0.9600
Cl5—C6	1.756 (6)	C8—H8A	0.9600
Cl6—C6	1.756 (5)	C8—H8B	0.9600
P1—O2	1.475 (3)	C8—H8C	0.9600
P1—O3	1.561 (4)	N3—C9	1.488 (6)
P1—O4	1.568 (4)	N3—C9^i^	1.488 (6)
P1—N1	1.598 (4)	N3—C10^i^	1.484 (6)
P2—O6	1.476 (3)	N3—C10	1.484 (7)
P2—O7	1.563 (4)	C9—H9A	0.9600
P2—O8	1.548 (5)	C9—H9B	0.9600
P2—N2	1.602 (5)	C9—H9C	0.9600
O1—C1	1.240 (6)	C10—H10A	0.9600
O3—C3	1.419 (7)	C10—H10B	0.9600
O4—C4	1.439 (6)	C10—H10C	0.9600
O5—C5	1.225 (5)		
			
O1^i^—La1—O1	138.20 (15)	Cl3A—C2—Cl2A	106.8 (5)
O2^i^—La1—O1^i^	71.11 (10)	C1—C2—Cl1A	111.3 (4)
O2^i^—La1—O1	143.90 (11)	C1—C2—Cl1B	117.3 (4)
O2—La1—O1^i^	143.90 (11)	C1—C2—Cl2A	105.2 (4)
O2—La1—O1	71.11 (10)	C1—C2—Cl2B	112.5 (4)
O2^i^—La1—O2	94.48 (15)	C1—C2—Cl3A	117.2 (4)
O2^i^—La1—O5	72.55 (11)	C1—C2—Cl3B	108.6 (4)
O2^i^—La1—O5^i^	77.50 (11)	O3—C3—H3A	109.5
O2—La1—O5^i^	72.55 (11)	O3—C3—H3B	109.5
O2—La1—O5	77.50 (11)	O3—C3—H3C	109.5
O2—La1—O6	141.22 (11)	H3A—C3—H3B	109.5
O2—La1—O6^i^	97.00 (12)	H3A—C3—H3C	109.5
O2^i^—La1—O6	97.00 (12)	H3B—C3—H3C	109.5
O2^i^—La1—O6^i^	141.22 (11)	O4—C4—H4A	109.5
O5—La1—O1^i^	125.40 (12)	O4—C4—H4B	109.5
O5—La1—O1	72.06 (12)	O4—C4—H4C	109.5
O5^i^—La1—O1^i^	72.06 (12)	H4A—C4—H4B	109.5
O5^i^—La1—O1	125.40 (12)	H4A—C4—H4C	109.5
O5^i^—La1—O5	135.31 (15)	H4B—C4—H4C	109.5
O6^i^—La1—O1^i^	78.01 (12)	O5—C5—N2	132.3 (5)
O6—La1—O1^i^	74.58 (12)	O5—C5—C6	113.6 (5)
O6^i^—La1—O1	74.58 (12)	N2—C5—C6	114.0 (4)
O6—La1—O1	78.01 (12)	Cl4—C6—Cl5	108.3 (3)
O6—La1—O5^i^	146.17 (12)	Cl4—C6—Cl6	108.0 (3)
O6^i^—La1—O5	146.17 (12)	Cl5—C6—Cl6	108.5 (3)
O6—La1—O5	70.98 (11)	C5—C6—Cl4	111.2 (3)
O6^i^—La1—O5^i^	70.98 (11)	C5—C6—Cl5	107.6 (4)
O6—La1—O6^i^	96.81 (16)	C5—C6—Cl6	113.2 (4)
O2—P1—O3	111.7 (2)	O7—C7—H7A	109.5
O2—P1—O4	106.12 (19)	O7—C7—H7B	109.5
O2—P1—N1	120.1 (2)	O7—C7—H7C	109.5
O3—P1—O4	106.0 (2)	H7A—C7—H7B	109.5
O3—P1—N1	103.4 (2)	H7A—C7—H7C	109.5
O4—P1—N1	108.8 (2)	H7B—C7—H7C	109.5
O6—P2—O7	106.9 (2)	O8—C8—H8A	109.5
O6—P2—O8	113.3 (2)	O8—C8—H8B	109.5
O6—P2—N2	118.6 (2)	O8—C8—H8C	109.5
O7—P2—N2	108.7 (3)	H8A—C8—H8B	109.5
O8—P2—O7	100.2 (3)	H8A—C8—H8C	109.5
O8—P2—N2	107.4 (3)	H8B—C8—H8C	109.5
C1—O1—La1	135.3 (3)	C9^i^—N3—C9	110.8 (6)
P1—O2—La1	136.36 (18)	C10—N3—C9^i^	108.2 (4)
C3—O3—P1	120.0 (4)	C10—N3—C9	109.9 (4)
C4—O4—P1	121.4 (4)	C10^i^—N3—C9^i^	109.9 (4)
C5—O5—La1	137.2 (3)	C10^i^—N3—C9	108.2 (4)
P2—O6—La1	136.72 (19)	C10^i^—N3—C10	110.0 (6)
C7—O7—P2	122.8 (4)	N3—C9—H9A	109.5
C8—O8—P2	120.3 (6)	N3—C9—H9B	109.5
C1—N1—P1	122.2 (3)	N3—C9—H9C	109.5
C5—N2—P2	123.4 (4)	H9A—C9—H9B	109.5
O1—C1—N1	132.9 (5)	H9A—C9—H9C	109.5
O1—C1—C2	113.9 (4)	H9B—C9—H9C	109.5
N1—C1—C2	113.2 (4)	N3—C10—H10A	109.5
Cl1A—C2—Cl2A	105.7 (5)	N3—C10—H10B	109.5
Cl1B—C2—Cl2B	106.9 (5)	N3—C10—H10C	109.5
Cl1B—C2—Cl3B	105.8 (5)	H10A—C10—H10B	109.5
Cl2B—C2—Cl3B	104.9 (5)	H10A—C10—H10C	109.5
Cl3A—C2—Cl1A	109.8 (5)	H10B—C10—H10C	109.5
			
La1—O1—C1—N1	17.5 (10)	O5—C5—C6—Cl6	154.3 (4)
La1—O1—C1—C2	−163.2 (3)	O6—P2—O7—C7	−171.5 (6)
La1—O5—C5—N2	10.5 (10)	O6—P2—O8—C8	63.4 (8)
La1—O5—C5—C6	−171.8 (3)	O6—P2—N2—C5	−6.4 (6)
P1—N1—C1—O1	−2.6 (9)	O7—P2—O6—La1	126.7 (3)
P1—N1—C1—C2	178.1 (3)	O7—P2—O8—C8	176.9 (8)
P2—N2—C5—O5	−0.1 (9)	O7—P2—N2—C5	−128.7 (5)
P2—N2—C5—C6	−177.8 (4)	O8—P2—O6—La1	−123.8 (3)
O1—C1—C2—Cl1A	32.5 (6)	O8—P2—O7—C7	70.2 (7)
O1—C1—C2—Cl1B	4.1 (7)	O8—P2—N2—C5	123.6 (5)
O1—C1—C2—Cl2A	−81.5 (6)	N1—P1—O2—La1	8.3 (4)
O1—C1—C2—Cl2B	−120.5 (6)	N1—P1—O3—C3	179.4 (5)
O1—C1—C2—Cl3A	160.0 (5)	N1—P1—O4—C4	−48.8 (5)
O1—C1—C2—Cl3B	123.9 (6)	N1—C1—C2—Cl1A	−148.0 (5)
O2—P1—O3—C3	−50.1 (6)	N1—C1—C2—Cl1B	−176.4 (5)
O2—P1—O4—C4	−179.4 (5)	N1—C1—C2—Cl2A	98.0 (6)
O2—P1—N1—C1	−10.0 (6)	N1—C1—C2—Cl2B	59.0 (7)
O3—P1—O2—La1	−112.9 (3)	N1—C1—C2—Cl3A	−20.5 (7)
O3—P1—O4—C4	61.8 (5)	N1—C1—C2—Cl3B	−56.6 (6)
O3—P1—N1—C1	115.2 (5)	N2—P2—O6—La1	3.5 (4)
O4—P1—O2—La1	132.1 (3)	N2—P2—O7—C7	−42.3 (7)
O4—P1—O3—C3	65.0 (6)	N2—P2—O8—C8	−69.6 (8)
O4—P1—N1—C1	−132.4 (4)	N2—C5—C6—Cl4	−149.4 (4)
O5—C5—C6—Cl4	32.5 (6)	N2—C5—C6—Cl5	92.3 (5)
O5—C5—C6—Cl5	−85.9 (5)	N2—C5—C6—Cl6	−27.5 (6)

Symmetry code: (i) −*x*+1/2, *y*, −*z*+3/2.

### Hydrogen-bond geometry (Å, º)

**Table:**

*D*—H···*A*	*D*—H	H···*A*	*D*···*A*	*D*—H···*A*
C9—H9*A*···O6	0.96	2.35	3.184 (7)	145
C10—H10*C*···O2^ii^	0.96	2.33	3.218 (8)	154

Symmetry code: (ii) −*x*+1/2, *y*+1, −*z*+3/2.
